# Research priorities for mitochondrial disorders: Current landscape and patient and professional views

**DOI:** 10.1002/jimd.12521

**Published:** 2022-05-31

**Authors:** Rhys H. Thomas, Amy Hunter, Lyndsey Butterworth, Catherine Feeney, Tracey D. Graves, Sarah Holmes, Pushpa Hossain, Jo Lowndes, Jenny Sharpe, Sheela Upadhyaya, Kristin N. Varhaug, Marcela Votruba, Russell Wheeler, Kristina Staley, Shamima Rahman

**Affiliations:** ^1^ Translational and Clinical Research Institute Newcastle University Newcastle UK; ^2^ Genetic Alliance UK London UK; ^3^ Lily Foundation Warlingham UK; ^4^ NHS Highly Specialised Service for Rare Mitochondrial Diseases, Newcastle Hospitals NHS Foundation Trust Newcastle UK; ^5^ Hinchingbrooke Hospital Huntingdon UK; ^6^ The National Hospital for Neurology and Neurosurgery London UK; ^7^ HCD Economics Ltd Warrington UK; ^8^ Oxford University Hospitals NHS Foundation Trust Oxford UK; ^9^ Centre for Innovation in Regulatory Science London UK; ^10^ James Lind Aliance Southampton UK; ^11^ University Hospital Wales and School of Vision Sciences Cardiff University Cardiff UK; ^12^ Leber's Hereditary Optic Neuropathy Society Hockley UK; ^13^ TwoCan Associates Herefordshire UK; ^14^ Mitochondrial Research Group, UCL Great Ormond Street Institute of Child Health and Great Ormond Street Hospital for Children NHS Foundation Trust London UK

**Keywords:** gene therapy, patient involvement, primary mitochondrial disease, priority setting partnership, treatment

## Abstract

Primary mitochondrial disorders encompass a wide range of clinical presentations and a spectrum of severity. They currently lack effective disease‐modifying therapies and have a high mortality and morbidity rate. It is therefore essential to know that competitively funded research designed by academics meets the core needs of people with mitochondrial disorders and their clinicians. Priority setting partnerships are an established collaborative methodology that brings patients, carers and families, charity representatives and clinicians together to try to establish the most pressing and unanswered research priorities for a particular disease. We developed a web‐based questionnaire, requesting all patients affected by primary mitochondrial disease, their carers and clinicians to pose their research questions. This yielded 709 questions from 147 participants. These were grouped into overarching themes including basic biology, causation, health services, clinical management, social impacts, prognosis, prevention, symptoms, treatment and psychological impact. Following the removal of “answered questions”, the process resulted in a list of 42 discrete, answerable questions. This was further refined by web‐based ranking by the community to 24 questions. These were debated at a face‐to‐face workshop attended by a diverse range of patients, carers, charity representatives and clinicians to create a definitive “Top 10 of unanswered research questions for primary mitochondrial disorders”. These Top 10 questions related to understanding biological processes, including triggers of disease onset, mechanisms underlying progression and reasons for differential symptoms between individuals with identical genetic mutations; new treatments; biomarker discovery; psychological support and optimal management of stroke‐like episodes and fatigue.

## 1 INTRODUCTION

Publicly funded and charitable research programmes demand accountability and favour translatable research. Clinical research, or research that may have clinical implications, therefore has a duty to know what impact the work and its conclusions may have for patients and their carers. “Nothing about me, without me” is the mantra of true patient partnership. Patients value and appreciate the potential advances that targeted research brings. This is particularly true in the mitochondrial disorders, where we currently lack effective disease‐modifying therapies and there is a very high morbidity and high mortality rate.

The primary mitochondrial disorders are a collection of varied multisystem conditions that share a single feature, namely that pathogenic variants (mutations) in DNA lead to disturbances of mitochondrial structure and function. Mutations may disrupt mitochondrial ultrastructure, impair the production of cofactors and vitamins, or disturb other metabolic processes within the mitochondrion, including the tricarboxylic acid cycle and pyruvate metabolism.^1^ However, it is clear that even for many of the more common mutations, our understanding of how they exert pathogenicity is incomplete.

The term mitochondrial disorder encompasses a broad number of clinical presentations and a spectrum of severity; clinical presentation can be from birth to late old age. Although recognisable patterns of clinical involvement can be seen, often depending on the pathogenic variant, there are considerable differences in symptoms between patients, between organ systems in the same patient, and across the life span. Accordingly, there is a need for multi‐disciplinary clinical teams to support these individuals and their families. The disorders are both individually and collectively rare and as such there are few clinicians, and fewer still academics, who are truly familiar with the challenges faced by people with mitochondrial disorders.

The James Lind Alliance (JLA) is a non‐profit making initiative established in 2004 that enables patients, carers and clinicians to work together in priority setting partnerships (PSPs). Previous successful PSPs range from ultra‐rare disorders such as rare inherited anaemias and dystrophic epidermolysis bullosa^2^ to much more common conditions including asthma, stroke, dementia and diabetes.^3^, ^4^, ^5^, ^6^ It is vital to know that competitively funded research designed by academics meets the core needs of people with mitochondrial disorders and their clinicians. We set out to follow the established methodology of the JLA to identify the Top 10 unanswered research priorities.^7^

## 2 METHODS

Genetic Alliance UK, supported by the Wellcome Trust, identified key charity and clinical partners to create a steering group which then grew through the suggestions of the initial members. Invitations to the steering group were targeted to promote strategic diversity, for example ensuring that the clinical members represented the geographic breadth of UK centres, differing clinical disciplines and both adult and paediatric patient groups (https://geneticalliance.org.uk/mitochondrial-diseases-psp-steering-group-members/). The views of patients were represented through four patient organisations: Leber's Hereditary Optic Neuropathy Society (LHON), The Lily Foundation, Metabolic Support UK and Muscular Dystrophy UK. Through consensus discussions, the steering group decided on the scope of this project. We included children *and* adults because of the life‐long nature of the disorders. Although diagnostic delays are important, they were thought to be pertinent to service delivery rather than research and so the focus of this PSP was on care, management, treatment, and natural history of the disorders rather than diagnosis. The focus of this PSP was limited to the primary mitochondrial disorders and not those where a secondary mitochondrial pathology may contribute to disease pathogenesis.

**FIGURE 1:**
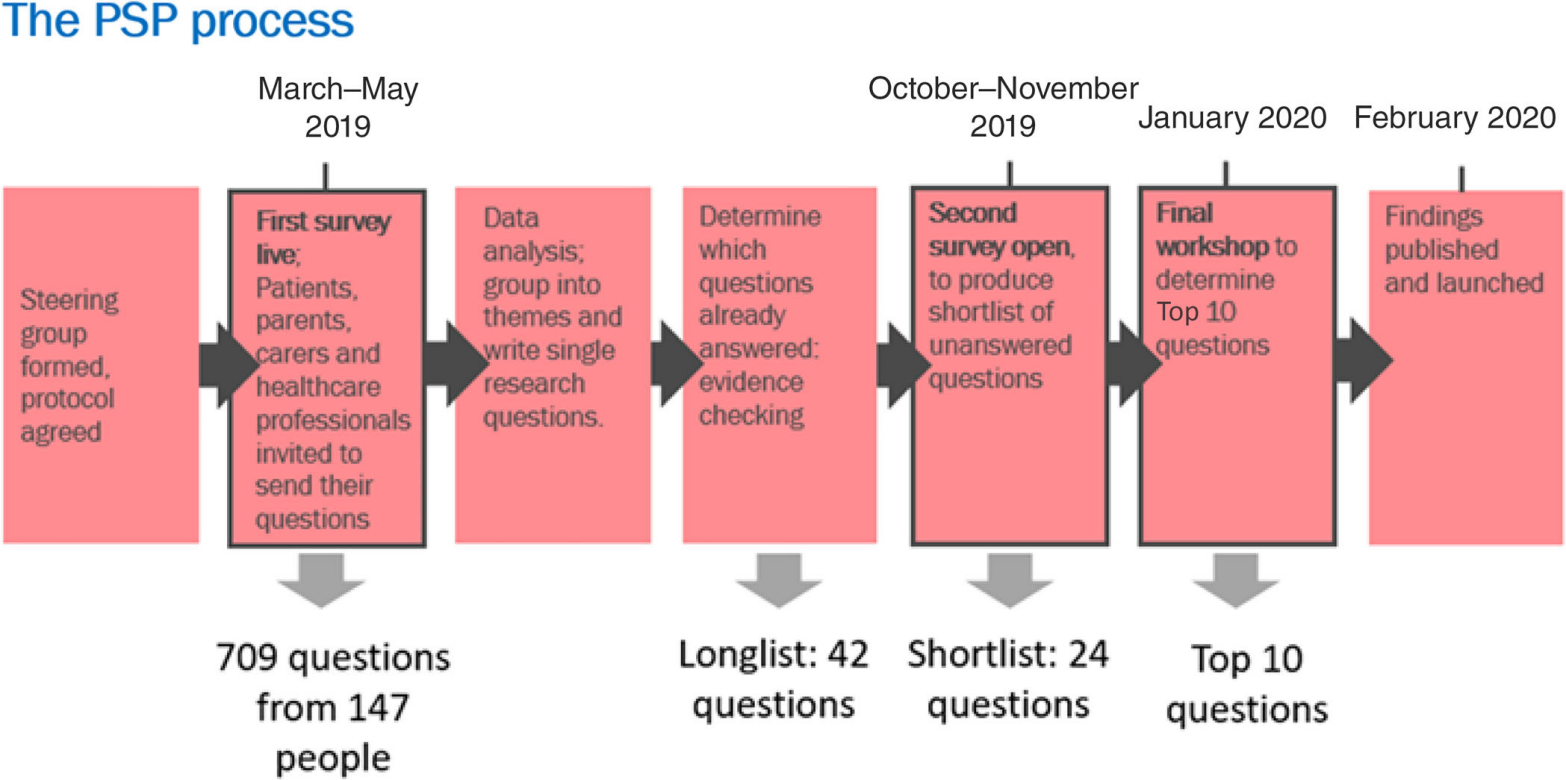
Flowchart illustrating the James Lind Alliance Priority Setting Partnership (PSP) process of gathering uncertainties, grouping them thematically, evidence checking, then analysing them before further reprioritisation and ranking to create a final “Top 10”

We followed the JLA framework, as this is a guided and reproducible process that is acknowledged to be the gold standard in research prioritisation by major research funding bodies (Figure 1).^8^ We first developed a web‐based questionnaire that was disseminated widely within UK networks, asking for all patients affected by primary mitochondrial disease, their carers and clinicians to pose their important unanswered research questions. Participation was encouraged by dissemination through local and national networks, including social media. At every stage, we actively sought to be inclusive of people with visual impairment and from minority ethnic groups. We produced videos in Arabic, Farsi, Malay and Welsh, a pragmatic choice given interpreters available in the timeframe, and used Facebook targeting to ensure that speakers of these languages saw the videos.

Questions were collated and cleaned, removing duplicate questions and suggestions that were not research questions but rather statements about personal experience. This long list of questions was then assembled into themes and similar questions were merged. Individually and in a face‐to‐face meeting, we analysed the question groupings and the wording of the merged umbrella questions written under each theme. No questions were removed because they could not be incorporated into a theme; a number of questions were taken forward to the next stage as single questions. The resulting 42 questions were then analysed to ensure that none had already been fully answered by existing published research or published guidelines. The search strategy used PUBMED as the primary source, limited to papers published in English and presenting human data (searches performed during August–September 2019). The secondary strategy utilised existing knowledge of “key papers” within the scientific literature by members of the steering group to identify the presence or absence of research in the areas highlighted by the questions. Following detailed literature searching, questions were declared “partially answered” or “unanswered”. No questions were declared “answered” and so none were removed from subsequent steps of the JLA process.

The 42 questions were then posed to patients, carers and clinicians in a second web‐based survey, asking them to rank these questions in terms of their importance. This generated a short list of 24 questions to be discussed at the final face‐to‐face workshop (Figure 1). At this final workshop 17 patients and carers, 2 representatives of patient organisations and 13 clinicians (25 of the 32 participants were independent of the steering group) ranked the questions using respectful debate and a “show of hands” where needed to gain consensus in three smaller working groups, each moderated by a JLA facilitator. A final plenary session ensured that the entire group reached agreement on the 10 questions and their ranking for the Top 10 research priorities for primary mitochondrial disease.

## 3 RESULTS

The initial process of asking the community for their research questions yielded 709 questions submitted by 50 patients, 47 carers and 50 clinicians. The process of thematic grouping, cleaning and evidence checking resulted in a list of 42 discrete, answerable questions (Table S1), which were ranked in the second web‐based survey. Eighteen of these questions were consistently ranked poorly by survey participants and were therefore excluded from further evaluation. The remaining 24 questions were taken forward to the final workshop (highlighted in bold in Table S1).

The 24 questions discussed at the final face‐to‐face workshop were grouped as members of certain categories. Some pertained to symptom control, such as “What is the most effective way to treat and manage pain?” Others asked about natural history, such as onset or progression of disease: “How does mitochondrial disease change over time as people get older?” Some questions were focussed on biological pathway discovery, for example “Could an understanding of the cellular and molecular processes in mitochondrial disease lead to new treatments?” After the ranking sessions, there was broad consensus regarding 9 of the Top 10 questions, and the 3 least valued questions, and the final plenary discussion resulted in unanimous agreement of a Top 10 (Table 1).

**TABLE 1:** The Top 10 unanswered research priorities for people with mitochondrial disorders

Top 10 priorities for research into mitochondrial disease
1. Could an understanding of the cellular and molecular processes in mitochondrial disease lead to new treatments?
2. Can the damage to cells caused by mitochondrial disease be repaired (e.g. to restore hearing, vision, or repair the pancreas)?
3. What are the biological mechanisms that cause mitochondrial disease to get worse over time?
4. What biomarkers (biological markers that can be measured, e.g. in blood samples) could be used to diagnose mitochondrial disease and to track its progress?
5. Could gene therapy help people with mitochondrial disease?
6. What are the psychological impacts of mitochondrial disease? What are the best ways to provide psychological support for people with mitochondrial disease and their families?
7. What are the best ways to reduce the risk of stroke‐like episodes in people with mitochondrial disease?
8. What factors could trigger the start of mitochondrial disease in people who have a genetic mutation?
9. Why are people with the same genetic mutation affected so differently in mitochondrial disease?
10. What are the most effective ways to treat and manage fatigue?

## 4 DISCUSSION: THE TOP 10 IN CONTEXT

What is known and/or how the area could be advanced for each of the Top 10 research priorities identified by the PSP:

Priority 1 *Could an understanding of the cellular and molecular processes in mitochondrial disease lead to new treatments?*

The highest‐ranking question addresses an urgent concern for all, the lack of disease specific treatment for the vast majority of people affected by mitochondrial disease. At the time of writing ClinicalTrials.gov recognises 175 interventional studies for people with “mitochondrial disease” (35 actively recruiting). The ambition in this question is central—although an increasing amount is known about mitochondrial biology, fundamental gaps in knowledge of mitochondrial disease pathogenesis remain and there are still almost no disease‐modifying therapies, the exception being coenzyme Q_10_ supplementation in some defects of coenzyme Q_10_ biosynthesis. It is still unknown whether a global treatment boosting mitochondrial function will be effective for a significant majority of patients or whether multiple gene‐specific personalised therapies are needed.^9^, ^10^

Priority 2 *Can the damage to cells caused by mitochondrial disease be repaired (e.g. to restore hearing, vision, or repair the pancreas)?*

The second‐ranked question is another appeal for novel therapeutics—and the hope for reparative therapy is that it could ameliorate disease pathology in those who are already symptomatic. There have been a number of attempts, such as the promising open label, non‐randomised clinical study Stem Cell Ophthalmology Treatment Study in LHON and autosomal dominant optic atrophy^11^, ^12^ but further work is needed in this area.

Priority 3 *Could gene therapy help people with mitochondrial disease?*

Some questions are broad and some narrow within the Top 10. Adeno‐associated viruses are commonly used vectors for gene therapy and have been trialled in animal models of nuclear DNA‐encoded mitochondrial diseases^13^, ^14^ but have not yet progressed to human clinical trials.^10^ The peculiarities of mitochondrial genetics mean that replicating this for mitochondrial DNA (mtDNA) has been more challenging, but a clinical trial success in LHON was reported recently^15^ and work is underway for *SURF1* associated Leigh syndrome.^16^ Another new development, which holds promise for new genetic therapies for mitochondrial disease, is the discovery of an effective method for base editing mtDNA.^17^

Priority 4 *What are the biological mechanisms that cause mitochondrial disease to get worse over time?*

The inherent processes that expedite or delay progression in mitochondrial disease are predominantly cryptic. For example, it was suggested that the combination of muscle mtDNA heteroplasmy level, mtDNA deletion size and the location of the mtDNA deletion within the mitochondrial genome predicted disease progression in adult patients with single, large‐scale mtDNA deletions (SLSMDs), but the same effects were not observed in a paediatric cohort with SLSMDs.^18^, ^19^ There remains a large unmet need for natural history studies that also collect biomarker data.

Priority 5 *What biomarkers (biological markers that can be measured* e.g. *in blood samples) could be used to diagnose mitochondrial disease and to track its progress?*

The biomarker question was seen to be essential to the delivery of high‐quality clinical trials and the development of therapy.^20^, ^21^ It is possible that answering this question will also address Priority 4. A number of putative biomarkers of mitochondrial dysfunction have been identified, including FGF21 and GDF15,^22^ but none of these is sufficiently specific and sensitive for all mitochondrial diseases.^21^ For mitochondrial disorders caused by mtDNA mutations, mtDNA heteroplasmy can in some cases be a clinically useful biomarker for assessment of disease and prognostication.^23^ However, no equivalent biomarker has been identified for nuclear‐encoded mitochondrial disease.

A number of questions focussed on specific impacts and symptoms.

Priority 6 *What are the psychological impacts of mitochondrial disease? What are the best ways to provide psychological support for people with mitochondrial disease and their families?*

This question was polarising, some rating it as most important and others as not being specific to mitochondrial disorders. Psychological support may include assisting families as many early‐onset mitochondrial disorders are associated with multisystem morbidity and significantly reduced life expectancy. There is also guilt and survivor's guilt that is associated with maternal transmission of some mitochondrial disorders.^24^ Participants in the PSP process also perceived this question as asking about specific psychological or psychiatric features of childhood, teenage and adult‐onset mitochondrial disorders.^25^ The importance of cognitive rehabilitation was also emphasised.^26^

Priority 7 *What are the best ways to reduce the risk of stroke‐like episodes in people with mitochondrial disease?*

This is an area of controversy, yet stroke‐like episodes contribute to anxiety before they occur and disability afterwards. There is no consensus for the acute treatment or prophylaxis against stroke‐like episodes. l‐arginine, l‐citrulline, taurine, l‐carnitine and coenzyme Q_10_ are used by some centres to treat stroke‐like episodes but have a weak evidence base.^27^ Anti‐epileptic drugs have been advocated in consensus guidelines.^28^ Current research is focussed on forecasting stroke‐like episodes in adults, dominated by people with the m.3243A>C mutation whereas much less is known about similar episodes in Leigh syndrome.^29^

Priority 8 *What are the most effective ways to treat and manage fatigue?*

Fatigue is an important issue that is commonly reported across PSPs; however, fatigue is a central and debilitating feature of many mitochondrial disorders.^30^ Fatigue management combined with gradated exercise programmes have been studied but optimum and effective treatments are still needed. Patients wanted support for both daily management of fatigue and also prevention of progressive symptom accumulation.

Priority 9 *What factors could trigger the start of mitochondrial disease in people who have a genetic mutation?*

This is a pertinent question to individuals who harbour a pathogenic mtDNA variant associated with LHON. A traditionally held view is that alcohol and tobacco are triggers for LHON.^31^ However, other authors have debated the importance of alcohol in isolation from smoking.^32^ Answering this question would open up preventative strategies for at risk individuals. Broadening this question beyond LHON would allow researchers to look at the broad phenotypic expression seen in *POLG*‐associated mitochondrial disease, and particularly those people in teenage years who may present de novo with seizures or stroke‐like episodes and whether there is a sex effect here.^33^

Priority 10 *Why are people with the same genetic mutation affected so differently in mitochondrial disease?*

At the evidence searching stage, it was concluded that the heteroplasmy level of mtDNA variants is not sufficient to answer this question since this fails to differentiate, for example, between the spectrum of disease seen in families with the m.3243A>G variant in the *MT‐TL1* gene.^34^ In some families, affected individuals predominantly have maternally inherited diabetes and deafness whilst in other families affected individuals may have mitochondrial encephalomyopathy with lactic acidosis and stroke‐like episodes. Both ostensibly have the same genetic cause, but the clinical manifestations vary considerably. Furthermore, some nuclear‐encoded mitochondrial disorders are also associated with remarkable phenotypic variability, even between individuals homozygous for the same genetic variant.^35^

### 4.1 Prioritisation process

We set out to engage patients, carers and clinicians with experience of mitochondrial disorders to pose research questions and rank them to create a shared Top 10 of unanswered research questions. Despite using the recognised PSP framework, we acknowledge limitations in our approach. There are challenges in gaining consensus about priorities in rare disease, particularly in the context of mitochondrial diseases, which are characterised by extreme complexity and heterogeneity. Pathogenic variants in approaching 400 different genes across two genomes may cause primary mitochondrial disease and affected individuals may experience symptoms related to dysfunction of virtually any organ in the body.^36^ Although contributions from more than 250 people with varied and valid lived experiences were secured in the PSP process, we could not possibly represent all the views that could be held by patients and carers. The PSP process, however, does not differentiate a research question on the basis of whether it was originally posed by one or many parties, favouring a process of ranking by consensus and retaining all asked questions visibly for researchers to scrutinise. We limited participation to those with a diagnosed disorder, potentially disadvantaging people in the early stages of their disease: it is challenging to engage people before they are diagnosed. The final face‐to‐face workshop involved group communication; although people with visual and auditory impairments participated, with appropriate support for their disabilities, no adults with intellectual impairment attended the workshop. Invitation to the final workshop was designed to primarily include clinicians and patients who were not part of the steering group. Care was taken to ensure that there was diversity in terms of the geographic, demographic and clinical characteristics of people at the workshop. No new priorities could be introduced at this workshop.

## 5 CONCLUSION

This model, a PSP, should be applicable for patients and clinicians who want to inform the research priorities for other rare diseases. It is unlikely that just because these priorities were identified in a UK setting that they would not be relevant in healthcare settings elsewhere. It may well be possible for people working in similar metabolic disorders to extrapolate priorities based upon those presented, although we would encourage genuine partnership and engagement if possible.

When using PSPs to set the research agenda, the Top 10s should be seen as a guide and not a mandate. It is in no one's best interest for patient prioritised research to unwittingly create conservative, risk‐free studies; there will always be a role for innovative and esoteric research. However, if funding bodies wish to reduce the risks of speculative research, they could consider supporting projects that also align with PSP priorities. In addition, it is advisable that all PSP Top 10s are reviewed and updated periodically. Finally, the process of producing Top 10s identifies unanswered questions, but is agnostic as to whether a question is answerable without significant technological advance.

The breadth of questions in our Top 10 research priorities brings out a number of themes. Some symptom‐based questions were prioritised, either because they are strikingly prominent across many mitochondrial disorders, such as fatigue, or because they present specific management challenges, such as stroke‐like episodes. Patients and clinicians were ambitious and calls for gene‐based therapies and treatments to reverse the process of neurodegeneration were valued highly. The call for better psychological support included identifying whether people with mitochondrial disease were more vulnerable to psychopathology, as well as appreciating the potent family dynamics of inherited progressive disease. The importance imbued on questions about biomarkers and identifying triggers spoke to a desire to engage more people at the pre‐symptomatic stage of their illness and to deliver disease modifying therapies to them as early as possible. The next step is for researchers, funders, and the pharmaceutical industry to react to the Top 10, and to align planned studies and funding criteria with them, as has been done after the publication of research priorities in other disease areas.

## AUTHOR CONTRIBUTIONS

Shamima Rahman was a member of the study steering committee, co‐wrote the article and is the guarantor. Rhys H. Thomas was clinical lead for the project, and drafted the article. Amy Hunter was patient lead for the project, and revised the article. Sheela Upadhyaya facilitated the project, and revised the article. Kristin N. Varhaug performed evidence checking during the project, and revised the article. Kristina Staley performed data analysis, and revised the article. Lyndsey Butterworth, Catherine Feeney, Tracey D. Graves, Sarah Holmes, Pushpa Hossain, Jo Lowndes, Jenny Sharpe, Marcela Votruba, and Russell Wheeler were members of the steering group and revised the article.

## CONFLICT OF INTERESTS

Rhys H. Thomas has received honoraria from Arvelle, Bial, Eisai, GW Pharma, Sanofi, UCB Pharma, UNEEG, Zogenix, and unrestricted research funding from Arvelle and UNEEG. Shamima Rahman sits on the scientific advisory board for Khondrion and the virtual scientific advisory boards for Pretzel, Taysha and Zogenix. She is the UK Chief Investigator for the MIT‐E trial (PTC Therapeutics), and investigator on the TEETPIM trial. She carries out consultancy activities with the following: BioMedical Insights, Epirium, Neurovive, Partners4Access, Pfizer, Stealth, Access Infinity. Marcela Votruba carries out consultancy activities with the following: Stoke Therapeutics, Santhera, Chiesi. She is an investigator on the LEROS study (Chiesi). Russell Wheeler has received honoraria from Santhera AG and Roche in the past. The LHON Society, for which Russell Wheeler is a trustee, has received sponsorship from Santhera AG, Gensight Biologics and Stealth Biotherapeutics.

## ETHICS STATEMENT

Ethics approval was not required. Informed consent was implicit in taking the online survey and attending the workshop.

## Supporting information


**TABLE S1** Draft summary questions and single questions

## Data Availability

All the research questions that were taken to the final workshop, and a downloadable document containing all questions generated by this project, are available on the James Lind Alliance website at the following link: https://www.jla.nihr.ac.uk/priority-setting-partnerships/mitochondrial-disease/top-10-priorities.htm
