# Supplementary material for: Research priorities for mitochondrial disorders: Current landscape and patient and professional views
Source: J Inherit Metab Dis. 2022 May 31;45(4):796–803. doi: 10.1002/jimd.12521 (PMC9429991; doi:10.1002/jimd.12521)
Supplement: Supplementary file 1 — TABLE S1 Draft summary questions and single questions [file JIMD-45-796-s001.docx]

| **Basic biology** | | |
| --- | --- | --- |
| **B1** | | **What are the genetic mutations that cause mitochondrial disease and how do they cause it?** |
| **B2** | | **Why are people with the same genetic mutation affected so differently in mitochondrial disease?** |
| **B3** | | **What are the biological mechanisms that cause mitochondrial disease to get worse over time?** |
| **B4** | | **What biomarkers (biological markers that can be measured e.g. in blood samples) could be used to diagnose mitochondrial disease and to track its progress?** |
| **B5** | | **How do the different genetic mutations cause the symptoms people experience with mitochondrial disease?** |
| **Cause** | | |
| **C1** | | **What causes the genetic mutation in people with mitochondrial disease whose parents don’t have the mutation?** |
| **C2** | | **What factors could trigger the start of mitochondrial disease in people who have a genetic mutation?** |
| **Health services** | | |
| **H1** | | How can health professionals (e.g. emergency doctors, GPs and hospital consultants) be properly informed about mitochondrial disease when they provide treatment and care? |
| **H2** | | **What level of monitoring of people with a mitochondrial disease is necessary to make sure they get the treatment and care they need at the right time? What aspects of their health should be monitored over time and how often?** |
| **H3** | | What are the best ways to organise care around the person with a mitochondrial disease? |
| **H5** | | What is the best end of life care for people with mitochondrial disease? |
| **Management** | | |
| **M1** | **Does exercise benefit people with mitochondrial disease? If yes, what is the best form of exercise?** | |
| **M2** | **Could a specific diet and/or supplements benefit people with mitochondrial disease?** | |
| **M3** | What lifestyle changes benefit people with mitochondrial disease (e.g. reducing stress, changing jobs, saunas)? | |
| **M4** | What can be learnt about managing the condition from people who successfully cope with mitochondrial disease, both mentally and physically? | |
| **Prognosis** | | |
| **P1** | **How does mitochondrial disease change over time as people get older?** | |
| **P2** | **Is there a way to predict who will become ill with mitochondrial disease, and whose symptoms will be worse?** | |
| **P3** | Are people with mitochondrial disease at greater risk of cancer? | |
| **P4** | How if life expectancy affected by mitochondrial disease? | |
| **P5** | How are males and females differently affected by mitochondrial disease? | |
| **P6** | What are the risks of children inheriting mitochondrial disease from their parents? | |
| **P7** | Are people with mitochondrial disease at greater risk from surgery and anaesthetic? | |
| **P8** | What are the risks of starving overnight e.g. before surgery or if unwell? | |
| **Prevention** | | |
| **R1** | **What can prevent the start of mitochondrial disease in people with a genetic mutation?** | |
| **R2** | **What can prevent mitochondrial disease from getting worse over time?** | |
| **Symptoms** | | |
| **S1** | **What are the most effective ways to treat and manage fatigue?** | |
| **S2** | **What are the most effective ways to treat and manage pain?** | |
| **S3** | **What are the most effective ways to treat and manage problems with memory, concentrating, learning and making decisions?** | |
| **S4** | **What are the most effective ways to treat and manage problems with balance and co-ordination?** | |
| **S5** | What are the most effective ways to treat and manage problems with eating and digestion? | |
| **S6** | **What are the most effective ways to treat and manage problems with muscle weakness?** | |
| **S7** | **What are the best ways to reduce the risk of stroke-like episodes in people with mitochondrial disease?** | |
| **Treatment** | | |
| **T1** | Do supplements prescribed by a doctor benefit people with mitochondrial disease? | |
| **T2** | **Could gene therapy help people with mitochondrial disease?** | |
| **T3** | Do alternative therapies benefit people with mitochondrial disease? | |
| **T4** | Would cannabinoid oil benefit people with mitochondrial disease? | |
| **T5** | Would routine physiotherapy benefit people with mitochondrial disease? | |
| **T6** | Why do some treatments work well for some people but not others? Does treatment need to be tailored to specific genetic mutations in mitochondrial disease? | |
| **T7** | **Can the damage to cells caused by mitochondrial disease be repaired (e.g. to restore hearing, or repair the pancreas)?** | |
| **T8** | Should the treatment of common conditions (e.g. diabetes and heart disease) be different in people with mitochondrial disease? | |
| **T9** | **Could an understanding of the cellular and molecular processes in mitochondrial disease lead to new treatments?** | |
| **Psychological impacts** | | |
| **Y1** | **What are the psychological impacts of mitochondrial disease? What are the best ways to provide psychological support for people with mitochondrial disease and their families?** | |
